# Salivary protein adsorption on titanium abutments: a comparative study of edentulous and partially edentulous patients

**DOI:** 10.3389/froh.2026.1827137

**Published:** 2026-06-09

**Authors:** Sharon Saldanha, Revathi P. Shenoy, Veena Hegde, Mahesh Mundathaje

**Affiliations:** 1 Department of Prosthodontics, Manipal College of Dental Sciences Mangalore, Manipal Academy of Higher Education, Manipal, India; 2 Department of Biochemistry, Kasturba Medical College, Manipal Academy of Higher Education, Manipal, India; 3 Department of Prosthodontics, Manipal College of Dental Sciences, Manipal Academy of Higher Education, Manipal, India

**Keywords:** edentulous, partially edentulous arch, protein adsorption, salivary proteins, titanium abutments

## Abstract

**Background:**

Salivary proteins rapidly adsorb onto titanium implant surfaces after placement, forming a pellicle that influences biofilm formation and peri-implant tissue responses. Differences in oral conditions between edentulous and partially edentulous patients may affect this protein adsorption and, consequently, peri-implant health.

**Methods:**

Sixteen adult patients (mean age: 51 years) who had implant therapy were categorized into edentulous and partially edentulous groups. One healing abutment per patient was randomly selected after intraoral exposure. Clinical parameters, including plaque index, were recorded. Adsorbed salivary proteins were identified using biochemical analysis and electrophoresis. Intergroup comparisons were performed using the Mann–Whitney *U* test, while associations between categorical variables were analyzed using Chi-square or Fisher's exact test where appropriate. Statistical significance was set at *p* ≤ 0.05.

**Results:**

Alpha-amylase, albumin, and immunoglobulin G (IgG) were consistently detected on titanium surfaces in both groups. No statistically significant difference was observed in the salivary protein profiles between edentulous and partially edentulous patients. However, plaque index demonstrated a statistically significant association with patient group (*p* < 0.05). The null hypothesis was therefore accepted, indicating no statistically significant intergroup difference in protein adsorption.

**Conclusion:**

The salivary pellicle formed on titanium implant components showed a similar protein composition in edentulous and partially edentulous patients. The significant association between plaque accumulation and patient group suggests that local oral hygiene conditions, rather than dentition status alone, may play a greater role in modulating the peri-implant biochemical environment, with potential implications for peri-implant inflammation and long-term implant success.

## 1 Introduction

Implant dentistry has transformed the rehabilitation of partially and completely edentulous patients by providing a predictable and widely accepted option for tooth replacement. Despite high reported success rates, dental implants remain susceptible to biological complications that may compromise long-term outcomes (1, 2).

The success of implant therapy relies on effective integration between artificial materials and the surrounding bone and soft tissues, and failures are commonly associated with surgical trauma, excessive occlusal loading, and microbial infection (3–6).

When implant components such as healing abutments are exposed to the oral environment, their surfaces are rapidly conditioned by saliva and plasma-derived cervicular fluid. This interaction leads to the formation of an acquired pellicle composed of selectively adsorbed salivary and serum-derived proteins, lipids, and other biomolecules. The acquired pellicle represents the earliest biological interface between the titanium surface and the oral environment. Unlike natural enamel, titanium exhibits distinct physicochemical properties, which may influence both the composition and functional behavior of the pellicle formed on its surface (7–9).

The acquired pellicle plays a critical role in early bacterial adhesion and subsequent biofilm development (3, 10, 11). Oral microorganisms interact with specific binding sites within pellicle proteins, initiating selective colonization of implant surfaces. The oral cavity harbors a highly diverse microbiota, with more than 700 bacterial species exhibiting site-specific colonization patterns (12). Certain planktonic bacteria possess adhesins that recognize pellicle-associated proteins such as alpha-amylase and proline-rich glycoproteins, facilitating attachment and biofilm maturation. Consequently, variations in pellicle composition may influence bacterial adhesion dynamics, biofilm architecture, and the progression of plaque-associated peri-implant diseases (13, 14).

Salivary composition and oral microbial ecology are modulated by several host-related factors, including dentition status. Partially edentulous and completely edentulous individuals differ in the presence of natural teeth, availability of plaque-retentive surfaces, mucosal characteristics, prosthesis design, and salivary flow patterns. These differences may alter the nature of salivary and serum protein adsorption onto implant abutment surfaces. However, evidence directly comparing the composition of the acquired pellicle on titanium surfaces between partially edentulous and edentulous patients under clinical conditions remains limited.

Most existing data on salivary protein adsorption to titanium surfaces are derived from *in vitro* studies using titanium discs exposed to pooled saliva or isolated protein fractions. Although these studies have demonstrated the adsorption of proteins such as alpha-amylase, albumin, and immunoglobulins, they do not adequately replicate the complex and dynamic oral environment encountered *in vivo*. As a result, the influence of patient-specific oral conditions on pellicle composition formed on implant components remains poorly understood (15–17).

To date, there is a lack of biochemical evidence evaluating whether dentition status affects the protein composition of the salivary pellicle formed on titanium implant components. This gap limits our understanding of how early biological conditioning of implant surfaces may contribute to microbial colonization and peri-implant inflammatory risk.

Therefore, this study aimed to identify and compare the salivary and serum-derived proteins adsorbed onto titanium abutment surfaces in partially edentulous and edentulous patients. The study sought to evaluate whether differences in dentition status influence the composition of the acquired pellicle and its potential clinical relevance in peri-implant disease development.

## 2 Subjects and methods

The research was carried out at the Department of Prosthodontics and Crown and Bridge of the Manipal College of Dental Sciences, Manipal, and the Department of Biochemistry of Kasturba Medical College, Manipal. The study protocol was conducted in accordance with the Declaration of Helsinki and approved by the KMC and KH Institutional Ethics Committee. Informed consent was obtained from all participants prior to their involvement in the study. This study was designed as a pilot, exploratory *in vivo* observational study with subsequent *ex vivo* biochemical analysis.

Using G*Power 3.1 software, the sample size was estimated based on an anticipated large effect size (Cohen's *d* = 1.2), derived from prior exploratory biomaterial and proteomic studies reporting substantial differences in protein adsorption patterns. With a two-tailed *t*-test, an alpha level of 0.05, and power (1 − *β*) of 0.80, the required sample size was calculated to be 7 participants per group. To accommodate potential variability and ensure balanced group allocation, 8 participants were included in each group. However, given the exploratory and pilot nature of the study, this sample size should be interpreted as preliminary, and the study is not powered for definitive inferential conclusions.

Adult patients undergoing stage-two implant surgery were eligible for inclusion if they had at least one titanium healing abutment in function for a minimum of 4 weeks. All participants were systemically healthy, non-smokers, and capable of providing unstimulated whole saliva. Individuals who had received antibiotic therapy or undergone professional oral prophylaxis within the preceding 4–6 weeks were excluded to minimize confounding effects on salivary protein composition.

Exclusion criteria included systemic conditions known to affect salivary flow or composition (such as Sjögren's syndrome and uncontrolled diabetes mellitus), current or past tobacco use, intake of medications known to alter salivary secretion (including anticholinergics, antidepressants, and *β*-blockers), presence of active periodontal or peri-implant disease, recent antibiotic use, and any condition that interfered with standardized saliva collection.

A total of 16 adult patients participated in this pilot study (mean age: 51 years), comprising 38% females and 62% males. Each participant contributed one implant site, from which a single titanium healing abutment was retrieved for analysis. A convenience sampling approach was used, enrolling 8 partially edentulous (*n* = 8) and 8 completely edentulous participants (*n* = 8) based on feasibility and availability during the study period.

All participants were rehabilitated using Nobel Replace Conical Connection titanium implants, characterized by an internal conical interface with internal hexagonal indexing, and restored with regular platform healing abutments during the healing phase. For partially edentulous patients, implants were placed in the posterior region, typically using diameters in the 4.3–5 mm range, with lengths spanning 8–13 mm. For completely edentulous patients, smaller diameters such as 3.5 mm were used, with comparable length options. The final prosthesis was screw retained.

### 2.1 Clinical examination

Plaque is an important etiological factor in peri-implantitis. To assess the clinical status of the peri-implant tissue in all 16 subjects, the periodontal parameter used was the plaque index by Lindquist et al. (18). Lindquist et al. evaluated oral hygiene using a three-point scale.

0: No visible plaque
1: Local plaque accumulation
2: Generalized plaque accumulation >25%

Calibration was performed using 10 pilot samples, yielding an inter-examiner reliability score (Cohen's kappa) >0.85. Standardized conditions (lighting, probing pressure, and scoring criteria) were maintained throughout data collection.

After exposure to the oral cavity for 4–6 weeks, the abutments were collected from each donor following a protocol approved by the Research and Ethics Committee. All samples were obtained under similar clinical conditions and processed using a uniform protocol, ensuring methodological consistency. To limit biological variability, all saliva-exposed abutments were retrieved during morning appointments between 9:00 and 11:00 a.m. Participants were instructed to refrain from eating, drinking (except water), or performing oral hygiene procedures for at least 2 h prior to sample collection. Unstimulated salivary flow rate was assessed chairside, and individuals with markedly reduced flow (<0.1 mL/min) were excluded to avoid atypical pellicle formation. Although diet was not otherwise controlled, all participants received standardized oral hygiene instructions 1 week before sampling to reduce inter-individual differences in plaque accumulation.

Protein extraction from titanium abutment surfaces was performed using a standardized elution protocol as follows: The samples were immediately placed in labeled plastic cryotubes containing phosphate-buffered saline (PBS) at pH 7. The cryotubes were then gently agitated for 1 min to detach all proteins adsorbed onto the titanium surfaces. The samples were clarified by centrifugation at 3800×*g*, 4 °C for 10 min. Later, the abutments were incubated at 37 °C with gentle rocking for 1 h. The abutments were then removed and rinsed twice with ultrapure Milli-Q water to eliminate loosely bound components. To remove the experimental pellicle from the abutment surfaces, the samples were treated with sodium dodecyl sulfate (SDS) sample buffer [Laemmli sample buffer, pH 6.8, 0.0625 M Tris (base), 2% SDS, 10% glycerin, 0.05 M DL-dithiothreitol (DL-DTT)] (19). This procedure effectively removes all proteins from the abutment surfaces, as confirmed by methods described by Vacca-Smith and Bowen (20). The samples were stored at −20 °C in a freezer for processing. This sequential extraction protocol ensured recovery of both loosely and strongly adsorbed salivary proteins from the titanium surface.

### 2.2 Blinding and bias control

To reduce bias, protein extraction, gel electrophoresis, and analysis were carried out by biochemistry laboratory personnel blinded to the clinical status (edentulous vs. partially edentulous) of the participants. Sample tubes were coded, and decoding was performed only after all analyses were completed.

Blinding of the clinical examiner to group assignment was not possible due to the obvious clinical distinction between edentulous and partially edentulous cases.

In cases where IgG was not detected, values were treated as absent and excluded from comparative statistical analysis where appropriate.

### 2.3 Total protein estimation

Total protein concentration was measured using the pyrogallol red-molybdate method, commonly employed for urine and cerebrospinal fluid protein estimation. A total protein reagent kit procured from Roche Diagnostics was used And Measured Using Cobas Integra Autoanalyzer (Cobas Integra from Roche Diagnostic Gmbh Germany).

Protein levels were measured using a colorimetric method based on pyrogallol red-molybdate complex. The pyrogallol red-molybdate (PRM) method is widely employed for quantifying total protein in samples such as urine and cerebrospinal fluid owing to its speed, affordability, and high sensitivity (21–24). The pyrogallol red–molybdate method was adapted from standard CSF protein quantification assays

When combined with molybdate, pyrogallol red forms a complex that absorbs light at a specific wavelength (460 nm). In an acidic medium, this complex binds to the basic amino acid groups of the proteins. This binding causes a shift in the absorption peak from 460 to 600 nm. The protein concentration was determined by measuring the increase in absorbance at 583 nm. The pyrogallol red-molybdate method is known for being simple, rapid, sensitive, and inexpensive. All procedures were performed under standardized laboratory conditions to minimize technical variability.

### 2.4 Amylase activity

Alpha-amylase activity was measured using a Hitachi 912 Autoanalyzer, with reagent kits sourced from Cobas Integra 400 (Roche Diagnostics GmbH, Germany). Alpha amylases facilitate the hydrolytic breakdown of polymeric carbohydrates, such as amylose, amylopectin, and glycogen, by cleaving *α*-1,4-glycosidic bonds in polysaccharides and oligosaccharides. The kinetic method relies on the cleavage of 4,6-ethylidine-(G7)-1,4-nitrophenyl-(G1)-*α*-D-maltoheptaoside by alpha-amylase, followed by the hydrolysis of all degradation products to *p*-nitrophenol using alpha-glucosidase. The color intensity of the resulting *p*-nitrophenol is directly proportional to alpha amylase activity and is measured spectrophotometrically.

### 2.5 IgG activity

The measurement is performed using the Immunoturbidimetric method with the COBAS INTEGRA Autoanalyzer (25). This instrument integrates four measurement principles: photometry, turbidimetry, ion-selective electrodes (ISE), and fluorescence polarimetry. It is a quantitative technique that determines the concentration of specific proteins by assessing the turbidity (cloudiness) resulting from antigen-antibody complex formation, which is directly proportional to the analyte concentration. This method relies on the principle that human IgG forms a precipitate with specific antiserum, which is turbidometrically measured at 340 nm.

### 2.6 Identification of albumin by polyacrylamide gel electrophoresis (page)

For the pellicle samples, SDS-PAGE was conducted following the Laemmli protocol (19) with standard procedures as described by Morrissey (26) and Vacca-Smith and Bowen (20).

SDS-PAGE was performed on the extracted experimental proteins obtained from 16 samples using the Laemmli method with a 12% acrylamide gel prepared in 0.025 M Tris-glycine buffer at pH 8.4. The stacking and running gels had pH values of 6.8 and 8.8, respectively. Samples were combined in a 2:1 ratio with a sample buffer containing 40% glycerol and 1.5 M Tris buffer (pH: 8.8) before loading onto the gel. Electrophoresis was performed at 80 V until the samples passed through the stacking gel, after which the voltage gradually increased to 200 V until the bromophenol blue dye front reached the end of the gel. Proteins were stained with 0.06% R-250 Coomassie Brilliant Blue and destained using a 3% acetic acid solution. Bovine serum albumin and lysozyme were used as molecular weight markers.

### 2.7 Statistical analysis

Statistical analysis was performed using IBM SPSS Statistics for Windows, version 25.0 (IBM Corp., Armonk, NY, USA). The distribution of continuous variables was assessed using the Shapiro–Wilk test. As the data did not meet assumptions of normality and given the small sample size, non-parametric tests were applied.

Intergroup comparisons between edentulous and partially edentulous patients were performed using the Mann–Whitney *U* test for continuous variables (total protein, amylase, and IgG levels), with results presented as median and interquartile range (IQR).

Categorical variables were analyzed using the Chi-square test or Fisher's exact test where appropriate. Plaque index was dichotomized into absence (score 0) and presence (scores 1 and 2) for statistical analysis.

Cases with missing IgG values due to insufficient sample volume were excluded from IgG-specific analysis.

A *p*-value ≤0.05 was considered statistically significant. Given the exploratory and pilot nature of the study and the limited sample size, findings should be interpreted with caution.

## 3 Results

**Figure 1:**
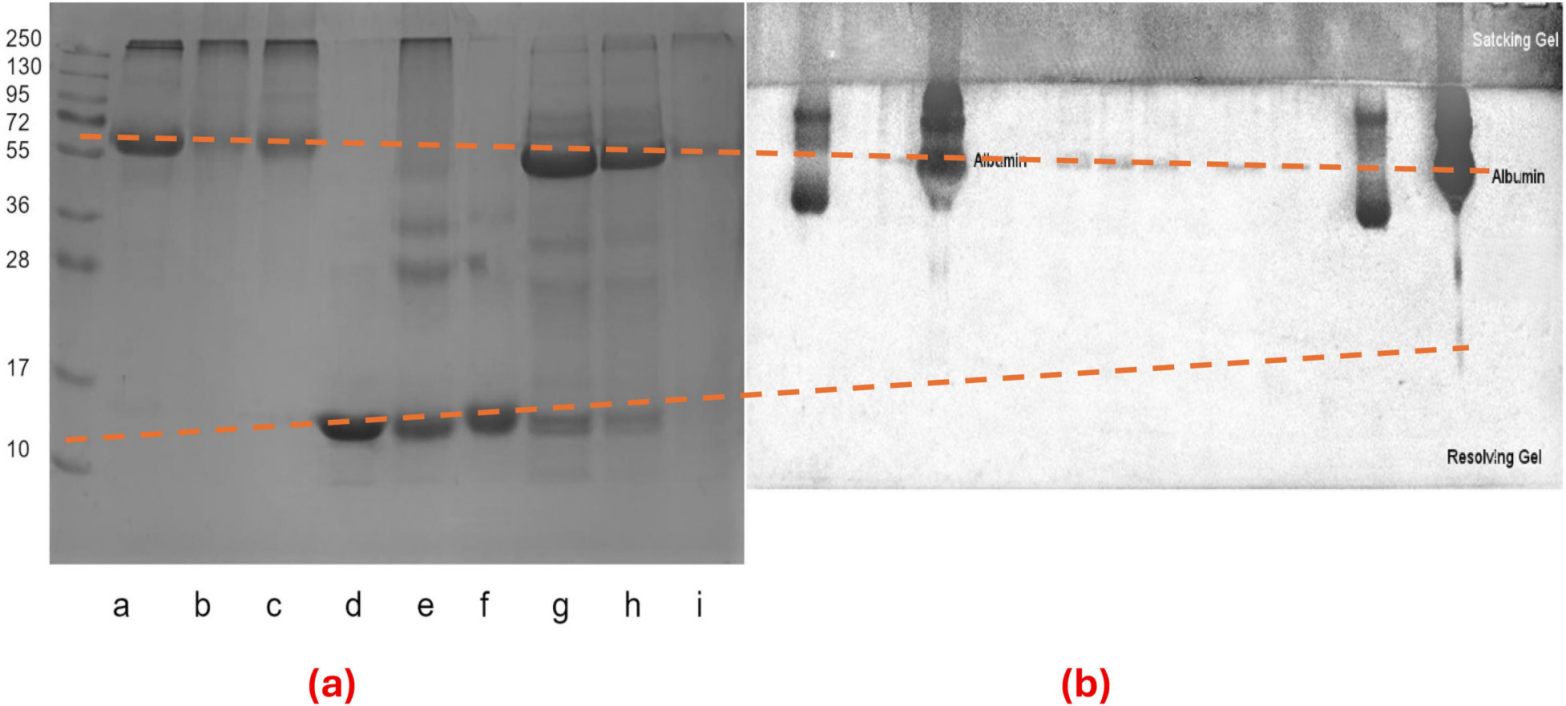
SDS–polyacrylamide gel electrophoresis (SDS-PAGE) profiles. (a) Protein profile of non-glycated BSA, lysozyme (LYS), and saliva, and samples glycated by fructose or methylglyoxal (MGO), adapted from Muraoka MY et al. (27). (b) Representative salivary protein banding patterns on titanium abutments in edentulous and partially edentulous patients. Bands corresponding approximately to α-amylase (∼55 kDa), albumin (∼66 kDa), and IgG (heavy chain ∼50 kDa; light chain ∼25 kDa) are indicated. Band identification was based on relative migration and comparison with published electrophoretic profiles. No distinct bands corresponding to lysozyme (∼14 kDa) were observed. Molecular weight estimation is approximate due to the absence of an internal marker.

A total of 16 participants were included, comprising 8 completely edentulous (*n* = 8) and 8 partially edentulous (*n* = 8) individuals. Detection of salivary proteins was performed using SDS–polyacrylamide gel electrophoresis. Distinct protein bands corresponding to the expected molecular weights of alpha-amylase (∼55 kDa), albumin (∼66 kDa), and immunoglobulin G (IgG; heavy chain ∼50 kDa, light chain ∼25 kDa) were observed in the salivary deposit samples (Figure 1b).

Based on molecular weight approximation and comparison with previously reported reference electrophoretic profiles reported by Muraoka et al. (27) Figure 1a, putative albumin was identified, while no discernible bands corresponding to the expected molecular weight of lysozyme (∼14 kDa) were observed in the salivary deposit samples from both edentulous and partially edentulous patients, as illustrated in Figure 1b. These observations represent qualitative assessment of protein banding patterns based on relative migration profiles.

Table 1 summarizes the demographic and biochemical characteristics of the study population, including total protein concentration, alpha-amylase activity, IgG activity, plaque index scores, and the presence or absence of albumin and lysozyme.

**Table 1:** Comparison of salivary biochemical and clinical parameters between edentulous and partially edentulous groups.

Edentulous patients (*n* = 8)	Protein (mg/dL)	Amylase (U/L)	IgG (mg/dL)	Albumin	Lysozyme	Plaque Index
E1	27	3,878	144	Present	Absent	0
E2	30	536	145	Present	Absent	2
E3	15	203	131	Present	Absent	2
E4	19	262	140	Present	Absent	1
E5	37	1,813	141	Present	Absent	2
E6	59	1,169	150	Present	Absent	1
E7	37	174	144	Present	Absent	0
E8	15	90	147	Present	Absent	1
P1	103	927	—	Present	Absent	0
P2	10	2295	138	Present	Absent	0
P3	26	535	144	Present	Absent	0
P4	45	8844	145	Present	Absent	0
P5	19	77	140	Present	Absent	0
P6	9	194	146	Present	Absent	0
P7	34	4427	144	Present	Absent	0
P8	8	37	—	Present	Absent	1

IgG values were unavailable for two partially edentulous participants due to insufficient sample volume; therefore, IgG analysis was performed on 14 samples.

PLAQUE INDEX.

0, No visible plaque.

1, Local plaque accumulation.

2, Generalised plaque accumulation > 25%.

The Mann–Whitney *U* test demonstrated no statistically significant differences in total protein concentration, alpha-amylase activity, or IgG activity between the groups (Table 2).

**Table 2:** Mann–Whitney *U* tests between the edentulous (1) and partially edentulous patients (2).

Ranks	Subject	*N*	Mean rank	Sum of ranks
Protein	1.00	8	9.31	79.00
	2.00	8	7.69	74.00
	Total	16		
AMY	1.00	8	8.25	66.00
	2.00	8	8.75	70.00
	Total	16		
IgG	1.00	8	7.75	62.00
	2.00	6	7.17	43.00
	Total	14		

IgG analysis included 14 samples (Edentulous = 8; Partially edentulous = 6). Two samples were excluded due to insufficient volume for reliable quantification.

The frequency distribution of plaque scores on titanium abutment surfaces is presented in Table 3. A plaque score of 0 was observed in 56.3% of abutments, a score of 1 in 25%, and a score of 2 in 18.7% of abutments.

**Table 3:** Distribution of plaque index scores between edentulous and partially edentulous subjects.

**Subject Group**	**Statistic**	**Plaque Index = 0**	**Plaque Index = 1**	**Plaque Index = 2**
Edentulous (1)	Count	2	3	3
	% within group	25.0%	37.5%	37.5%
Partially Edentulous (2)	Count	7	1	0
	% within group	88.9%	11.1%	0%
**Total**	Count	9	4	3
	% within group	56.3%	25.0%	18.7%

Association between plaque index scores and dentate status was assessed using the Chi-square test, with Fisher's exact test applied where appropriate. A statistically significant association was identified between plaque index and patient group (*p* = 0.015) detailed in Table 4. These findings should be interpreted within the constraints of the study design and reflect observed associations rather than causal relationships. Missing data points (e.g., IgG bands not detected in certain samples) were treated as absent and excluded from quantitative comparison where applicable.

**Table 4:** Association between plaque index and subject group (Chi-square test).

**Test**	**Value**	**df**	**Asymptotic Significance (2-sided)**	**Exact Significance (2-sided)**	**Exact Significance (1-sided)**
Pearson Chi-Square	7.137	1	0.008	—	—
Continuity Correction	4.743	1	0.029	—	—
Likelihood Ratio	7.758	1	0.005	—	—
Fisher’s Exact Test	—	—	—	**0.015**	**0.013**
Linear-by-Linear Association	6.717	1	0.010	—	—
N of Valid Cases	17	—	—	—	—

A statistically significant association was observed between subject group and plaque index distribution (Pearson Chi-square, *p* = 0.008). This finding was consistent across alternative tests, including Fisher’s exact test (*p* = 0.015), confirming the robustness of the result

## 4 Discussion

Protein bands corresponding to molecular weights of alpha-amylase, albumin, and IgG were observed, suggesting their likely presence. However, it should be noted that SDS-PAGE provides presumptive identification based on molecular weight and does not confirm protein identity. The proteins recognized in our experimental pellicles were consistent with those reported by Vacca-Smith and Bowen (20). No statistically significant difference was noted in the salivary deposit sample content between the edentulous and partially edentulous patients. This similarity likely reflects a common adsorption pattern on titanium within the oral environment. This finding is biologically plausible, as titanium surfaces are covered by a highly reactive oxide layer that promotes nonspecific adsorption of salivary and serum proteins, irrespective of dentition status.

Salivary pellicles are known to contain proteins such as amylase, proline-rich proteins, cystatins, and immunoglobulins, many of which have been identified on implant abutment surfaces. Implant and restorative materials possess surface characteristics distinct from natural enamel, with modern micro and nano scale modifications along with changes in surface energy, it can influence pellicle formation by altering early protein adsorption kinetics and thus result in a biological environment conducive for bacterial adhesion and colonization (9, 28, 29). The detection of albumin, alpha-amylase, and IgG in both groups supports the concept of selective protein adsorption onto titanium surfaces (30). Studies have demonstrated that surface-modified titanium abutments exhibit distinct protein adsorption profiles compared to machined titanium, with surface topography and surface energy playing a critical role in determining protein conformation and biological activity (31–33).

The consistent detection of albumin, alpha-amylase, and IgG in both groups supports selective protein adsorption onto titanium, suggesting preferential affinity of specific salivary molecules.

Inter-individual variability in albumin adsorption suggests patient-specific differences in salivary composition. Although both albumin and lysozyme are secreted by the major salivary glands, only albumin was detected in the present PAGE analysis. Lysozyme was not detected in any sample. This may be attributed to selective adsorption phenomena, competitive displacement by higher-molecular-weight proteins such as albumin, or reduced retention during pellicle maturation. However, these mechanisms were not directly evaluated in the present study (34, 35). Definitive protein identification would require confirmatory techniques such as Western blotting or mass spectrometry.

Similar protein adsorption patterns have been reported *in vitro* and *in vivo* studies evaluating salivary pellicle formation on contemporary implant abutment materials (36–38). This selective adsorption of proteins to titanium may explain both its integration with bone via its capacity to bond to blood proteins and reduced plaque formation compared to enamel (39–41). Alpha-amylase identified in the present samples has also been reported in enamel pellicles, where its adsorption is considered less persistent than that of several other salivary proteins (34, 42).

Plaque around the implant abutments was evaluated using the modified plaque index by Lindquist et al. (18). Despite the availability of newer peri-implant–specific indices, modified plaque indices continue to be widely used in clinical implant research for longitudinal assessment of plaque accumulation (43–45). In the edentulous group, 75% of subjects exhibited plaque index scores of 1 or 2, whereas 88.9% of partially edentulous patients demonstrated a plaque index score of 0. Overall, plaque accumulation was higher in edentulous patients; however, potential contributing factors such as peri-abutment tissue conditions, oral hygiene, dietary habits, and abutment location were not evaluated, and the findings should therefore be interpreted cautiously. Overall, plaque accumulation trends were consistent with previous clinical observations; however, the differences between groups should be interpreted as associative rather than causal (46–50). However, no mechanistic relationship between protein adsorption and plaque accumulation was evaluated in this study.

Differences in protein adsorption on titanium abutment surfaces may have important implications for peri-implant disease susceptibility, as the initial protein layer forms the biological interface that governs subsequent bacterial adhesion and host responses. Importantly, the physicochemical properties of titanium, including surface energy and oxide layer composition, may override patient-related variables in determining early pellicle formation. Adsorbed salivary proteins such as albumin, alpha-amylase, and immunoglobulins can modulate early biofilm formation by selectively promoting or inhibiting bacterial attachment (14, 51, 52). Alpha-amylase is known to facilitate the adhesion of oral streptococci by acting as a binding substrate, potentially enhancing early plaque accumulation on implant surfaces (53). Albumin, while often associated with passive adsorption, can alter surface wettability and influence the composition and maturation of the acquired pellicle, thereby indirectly affecting microbial colonization (54). IgG present on implant surfaces may reflect host immune activity and could participate in opsonization processes; however, its effectiveness in preventing bacterial colonization on abiotic surfaces remains uncertain (55). Variations in the quantity or composition of these adsorbed proteins may therefore shift the balance between a stable peri-implant environment and conditions favoring plaque accumulation and inflammation (56). In the presence of increased plaque retention, these protein-mediated interactions may have potential relevance to peri-implant biological processes; however, this relationship was not directly investigated in the present study. Nevertheless, given the observational nature of the present study, these associations should be interpreted cautiously, and causal relationships cannot be inferred.

### 4.1 Limitations

Several methodological limitations should be considered when interpreting these findings. The relatively small sample size and pilot nature of the study limit statistical power and generalizability, and the use of convenience sampling may introduce selection bias. The cross-sectional design further precludes causal inferences. The absence of a control group (e.g., unused abutments or baseline surface analysis) restricts the ability to differentiate between pre-existing and acquired protein adsorption. Additionally, protein identification was based on SDS-PAGE and therefore remains presumptive, as this technique relies on molecular weight estimation rather than definitive characterization. In addition, the absence of an internal molecular weight marker within the electrophoretic gel and reliance on molecular weight approximation using previously published reference profiles, including those reported by Muraoka MY et al. (27), may further limit the precision of band identification and interpretation. A detailed physicochemical characterization of the titanium abutment surfaces was not performed. Since surface properties such as roughness, charge, and surface energy influence protein adsorption, this limits mechanistic interpretation of the findings. Potential confounding variables including dietary habits, salivary composition, flow rate, circadian variation, implant location, and oral hygiene practices were not controlled and may have contributed to variability in protein adsorption and plaque scores. Furthermore, while abutments were exposed *in vivo*, subsequent biochemical analysis was conducted *ex vivo*, which may not fully capture dynamic biological interactions over time. Finally, no microbiological correlation was performed, and the molecular pathways linking protein adsorption to bacterial adhesion and peri-implant disease were not directly investigated. Additionally, the absence of advanced proteomic techniques such as mass spectrometry limits definitive protein characterization. Longitudinal studies integrating biomolecular and clinical outcomes are therefore warranted.

## 5 Conclusions

Within the scope of this study, titanium abutment surfaces in both edentulous and partially edentulous patients demonstrated selective adsorption of salivary proteins, predominantly alpha-amylase, albumin, and immunoglobulin G. The consistency of this protein profile across groups suggests the presence of a common biological adsorption process on titanium within the oral environment.

Because the acquired pellicle forms the initial interface between biomaterials and host tissues, these early protein deposits may influence subsequent microbial attachment and peri-implant biofilm development. Although bacterial colonization was not directly evaluated, the findings highlight protein adsorption as a potentially important upstream event in shaping peri-implant ecology.

A deeper understanding of these early biomolecular interactions may support the future design of implant surfaces that promote biologically favorable host–microbial relationships. Further longitudinal *in vivo* studies integrating proteomic and microbiological analyses are necessary to clarify the clinical implications of these observations.

## Data Availability

The original contributions presented in the study are included in the article/Supplementary Material, further inquiries can be directed to the corresponding author.

## References

[1] Tomasi C Derks J. Etiology, occurrence, and consequences of implant loss. Periodontol 2000. (2022) 88:13–35. 10.1111/prd.12408 35103324 PMC9306999

[2] Derks J Schaller D Håkansson J Wennström JL Tomasi C Berglundh T. Peri-implantitis—onset and pattern of progression. J Clin Periodontol. (2016) 43:383–388. 10.1111/jcpe.12535 26900869

[3] Manaf J Rahman S Haque S Alam M. Bacterial colonization and dental implants: a microbiological study. Pesqui Bras Odontopediatr Clin Integr. (2020) 20:e0105. 10.1590/pboci.2020.105

[4] Schwarz F Jepsen S Obreja K Galarraga-Vinueza ME Ramanauskaite A. Surgical therapy of peri-implantitis. Periodontol 2000. (2022) 88:145–181. 10.1111/prd.12417 35103328

[5] Ramanauskaite A Schwarz F Sader R. Influence of width of keratinized tissue on the prevalence of peri-implant diseases: a systematic review and meta-analysis. Clin Oral Implants Res. (2022) 33(Suppl 23):8–31. 10.1111/clr.13766 35763022

[6] Lindhe J Meyle J, Group D of the European Workshop on Periodontology. Peri-implant diseases: consensus report of the sixth European workshop on periodontology. J Clin Periodontol. (2008) 35(Suppl 8):282–285. 10.1111/j.1600-051X.2008.01283.x 18724855

[7] Hannig C Hannig M Kensche A Carpenter G. The mucosal pellicle—an underestimated factor in oral physiology. Arch Oral Biol. (2017) 80:144–152. 10.1016/j.archoralbio.2017.04.001 28419912

[8] Siqueira WL Custodio W McDonald EE. New insights into the composition and functions of the acquired enamel pellicle. J Dent Res. (2012) 91(12):1110–1118. 10.1177/0022034512462578 23018818

[9] Barberi J Spriano S. Titanium and protein adsorption: an overview of mechanisms and effects of surface features. Materials. (2021) 14(7):1590. 10.3390/ma14071590 33805137 PMC8037091

[10] Martínez-Hernández M Reyes-Grajeda JP Hannig M Almaguer-Flores A. Salivary pellicle modulates biofilm formation on titanium surfaces. Clin Oral Investig. (2023) 27(10):6135–6145. 10.1007/s00784-023-05230-9 PMC10560156 37646908

[11] Lima EM Koo H Vacca Smith AM Rosalen PL Del Bel Cury AA. Adsorption of salivary and serum proteins and bacterial adherence on titanium and zirconia ceramic surfaces. Clin Oral Implants Res. (2008) 19(8):780–785. 10.1111/j.1600-0501.2008.01524.x 18705809

[12] Donlan RM. Biofilms: microbial life on surfaces. Emerg Infect Dis. (2002) 8(9):881–890. 10.3201/eid0809.020063 12194761 PMC2732559

[13] Gibbons RJ van Houte J. Bacterial adherence and the formation of dental plaques. In: Beachey EH, editor. Bacterial Adherence. Receptors and Recognition. Dordrecht: Springer (1980). p. 61–104. 10.1007/978-94-009-5863-0_3

[14] Quirynen M Bollen CM. The influence of surface roughness and surface-free energy on supra- and subgingival plaque formation in man. J Clin Periodontol. (1995) 22(1):1–14. 10.1111/j.1600-051X.1995.tb01765.x 7706534

[15] Kolenbrander PE Palmer RJ Periasamy S Jakubovics NS. Oral multispecies biofilm development and the key role of cell–cell distance. Nat Rev Microbiol. (2010) 8(7):471–480. 10.1038/nrmicro2381 20514044

[16] Koo H Diaz PI Kreth J. The functional oral microbiome: biofilm environment, polymicrobial interactions, and community dynamics. Mol Oral Microbiol. (2022) 37(5):165–166. 10.1111/omi.12390 36169983

[17] Matthes de Freitas Pontes K Fontenelle IS Nascimento CD Oliveira VC Albuquerque Garcia B Silva PGB. Clinical study of the biofilm of implant-supported complete dentures in healthy patients. Gerodontology. (2022) 39(2):148–160. 10.1111/ger.12547 33660315

[18] Lindquist LW Rockler B Carlsson GE. Bone resorption around fixtures in edentulous patients treated with mandibular fixed tissue-integrated prostheses. J Prosthet Dent. (1988) 59(1):59–63. 10.1016/0022-3913(88)90109-6 3422305

[19] Laemmli UK. Cleavage of structural proteins during the assembly of the head of bacteriophage T4. Nature. (1970) 227(5259):680–685. 10.1038/227680a0 5432063

[20] Vacca Smith AM Bowen WH. The effects of milk and kappa-casein on salivary pellicle formed on hydroxyapatite discs in situ. Caries Res. (2000) 34(1):88–93. 10.1159/000016558 10601790

[21] Orsonneau JL Douet P Massoubre C Lustenberger P Bernard S. An improved pyrogallol red-molybdate method for determining total urinary protein. Clin Chem. (1989) 35(11):2233–2236. 10.1093/clinchem/35.11.2233 2582622

[22] Phillipou G James SK Seaborn CJ Phillips PJ. Screening for microalbuminuria by using a rapid, low-cost colorimetric assay. Clin Chem. (1989) 35(3):456–458. 10.1093/clinchem/35.3.456 2920413

[23] Watanabe N Kamei S Ohkubo A Yamanaka M Ohsawa S Makino K. Urinary protein as measured with a pyrogallol red-molybdate complex, manually and in a hitachi 726 automated analyzer. Clin Chem. (1986) 32(8):1551–4. 10.1093/clinchem/32.8.1551 3731450

[24] Van Ingen HE. Automated analysis for urinary protein by pyrogallol red–molybdate method. Clin Chem. (1990) 36(4):702. 10.1093/clinchem/36.4.702a 2323054

[25] Sánchez A Mirabel JL Barrenechea E Eugui J Puelles A Castañeda A. Evaluation of an improved immunoturbidimetric assay for serum C-reactive protein on a COBAS INTEGRA 400 analyzer. Clin Lab. (2002) 48(5–6):313–7. 12071582

[26] Morrissey JH. Silver stain for proteins in polyacrylamide gels: a modified procedure with enhanced uniform sensitivity. Anal Biochem. (1981) 117(2):307–310. 10.1016/0003-2697(81)90783-1 6172996

[27] Muraoka MY Justino AB Caixeta DC Queiroz JS Sabino-Silva R Salmen Espindola F. Fructose and methylglyoxal-induced glycation alters structural and functional properties of salivary proteins, albumin and lysozyme. PLoS One. (2022) 17(1):e0262369. 10.1371/journal.pone.0262369 35061788 PMC8782344

[28] Stich T Alagboso F Křenek T Kovářík T Alt V Docheva D. Implant-bone interface: reviewing the impact of titanium surface modifications on osteogenic processes *in vitro* and *in vivo*. Bioeng Transl. (2022) 7(1):e10239. 10.1002/btm2.10239 PMC8780039 35079626

[29] Rodrigues E Almeida T Oliveira F Albergaria J Ghosh S Tavares M. Comparative study of nanostructured TiO_2_ and SLA surface modifications for titanium implants: surface morphology and *in vitro* evaluation. Mater Res. (2022) 25:e20210613. 10.1590/1980-5373-mr-2021-0613

[30] Carlén A Nikdel K Wennerberg A Holmberg K Olsson J, Surface characteristics and in vitro biofilm formation on glass ionomer and composite resin. Biomaterials. (2001) 22:481–7. 10.1016/S0142-9612(00)00204-0 11214759

[31] Williams DF Askill IN Smith R. Protein absorption and desorption phenomena on clean metal surfaces. J Biomed Mater Res. (1985) 19(3):313–20. 10.1002/jbm.820190312 4077885

[32] Łosiewicz B Osak P Nowińska D Maszybrocka J. Developments in dental implant surface modification. Coatings. (2025) 15(1):109. 10.3390/coatings15010109

[33] Kunrath MF Dahlin C. The impact of early saliva interaction on dental implants and biomaterials for oral regeneration: an overview. Int J Mol Sci. (2022) 23(4):2024. 10.3390/ijms23042024 35216139 PMC8875286

[34] Bower RC Radny NR Wall CD Henry PJ. Clinical and microscopic findings in edentulous patients, three years after incorporation of osseointegrated implant-supported bridgework. J Clin Periodontol. (1989) 16(9):580–587. 10.1111/j.1600-051X.1989.tb02141.x 2794093

[35] Ostry D Kraus FW. Immunofluorescent demonstration of specific proteins in human early dental enamel. J Oral Pathol. (1973) 2(2):68–76. 10.1111/j.1600-0714.1973.tb01675.x 4133118

[36] Wu Y Wan K Lu J Yuan C Cui Y Duan R. Research progress on surface modification of titanium implants. Coatings. (2025) 15(2):229. 10.3390/coatings15020229

[37] Sarfraz S. Bacterial adhesion on patient-specific implant materials (doctoral dissertation on the Internet). University of Oulu, Oulu, FI (2024). (Accessed February 16, 2026).

[38] Martínez-Hernández M Reyes-Mendoza P Chávez-Esparza M Rodríguez-Hernández AP García-Pérez VI. The modulating effect on *Staphylococcus species* and *Pseudomonas aeruginosa* biofilm development of salivary pellicle conditioning titanium surfaces. Res Sq [Preprint]. (2025). 10.21203/rs.3.rs-6638066/v1 PMC13469995 42236858

[39] Banakar M Wei L. Bio-Based Nanomaterials in Dentistry. Cham: Springer Nature (2024).

[40] Kohavi D Klinger A Steinberg D Sela MN. Adsorption of salivary proteins onto prosthetic titanium components. J Prosthet Dent. (1995) 74(5):531–534. 10.1016/s0022-3913(05)80357-9 8809261

[41] Steinberg D Klinger A Kohavi D Sela MN. Adsorption of human salivary proteins to titanium powder. I. Adsorption of human salivary albumin. Biomaterials. (1995) 16(17):1339–43. 10.1016/0142-9612(95)91050-9 8573673

[42] Edgerton M Levine MJ. Characterization of acquired denture pellicle from healthy and stomatitis patients. J Prosthet Dent. (1992) 68(4):683–691. 10.1016/0022-3913(92)90387-P 1403950

[43] Mombelli A Lang NP. Clinical parameters for the evaluation of dental implants. Periodontol 2000. (1994) 4(1):81–86. 10.1111/j.1600-0757.1994.tb00008.x 9673196

[44] Sabri H Tavelli L Sheikh AT Kalani K Zimmer JM Barootchi S. Significance of peri-implant keratinised mucosa on implant health: an umbrella systematic review with evidence mapping and quantitative meta-meta-analysis. Int J Oral Implantol. (2025) 18(1):13–30. 40047360

[45] Herrera D Berglundh T Schwarz F Chapple I Jepsen S Sculean A. Prevention and treatment of peri-implant diseases—the EFP S3 level clinical practice guideline. J Clin Periodontol. (2023) 50(Suppl 26):4–76. 10.1111/jcpe.13823 37271498

[46] Derks J Ichioka Y Dionigi C Trullenque-Eriksson A Berglundh J Tomasi C. Prevention and management of peri-implant mucositis and peri-implantitis: a systematic review of outcome measures used in clinical studies in the last 10 years. J Clin Periodontol. (2023) 50(Suppl 26):55–66. 10.1111/jcpe.13608 35246865

[47] Lekholm U Adell R Lindhe J Brånemark PI Eriksson B Rockler B. Marginal tissue reactions at osseointegrated titanium fixtures (II): a cross-sectional retrospective study. Int J Oral Maxillofac Surg. (1986) 15(1):53–61. 10.1016/S0300-9785(86)80011-4 3083006

[48] Ong ES Newman HN Wilson M Bulman JS. The occurrence of periodontitis-related microorganisms in relation to titanium implants. J Periodontol. (1992) 63(3):200–205. 10.1902/jop.1992.63.3.200 1317427

[49] Apse P Ellen RP Overall CM Zarb GA. Microbiota and crevicular fluid collagenase activity in the osseointegrated dental implant sulcus: a comparison of four sites in edentulous and partially edentulous patients. J Periodontal Res. (1989) 24(2):96–105. 10.1111/j.1600-0765.1989.tb00863.x 2542514

[50] Danser MM Van Winkelhoff AJ Van der Velden U. Periodontal bacteria colonising oral mucous membranes in edentulous patients wearing dental implants. J Periodontol. (1997) 68(3):209–216. 10.1902/jop.1997.68.3.209 9100195

[51] Lavelle CLB. Applied Physiology of the Mouth, Salivary Glands and Saliva. London: Wright (1975). p. 145–149.

[52] Rechendorff K Hovgaard M Foss M Zhdanov V Besenbacher F. Enhancement of protein adsorption induced by surface roughness. Langmuir. (2007) 22:10885–8. 10.1021/la0621923 17154557

[53] Sabharwal A Haase EM Scannapieco FA. Amylase binding to oral streptococci: a key interaction for human oral microbial ecology, adaptation and fitness. Biomolecules. (2025) 15(11):1616. 10.3390/biom15111616 41301533 PMC12650345

[54] Le H Droniou M Wallart L Coquet L Thebault P Guillou C. Pre-adsorption of serum albumin on biomaterial surfaces modulates bacteria-surface interactions and alters bacterial physiological responses. Mater Today Bio. (2025) 35:102254. 10.1016/j.mtbio.2025.102254 41625360 PMC12859551

[55] Brandquist ND Kielian T. Immune dysfunction during S. aureus biofilm-associated implant infections: opportunities for novel therapeutic strategies. npj Biofilms Microbiomes. (2025) 11:144. 10.1038/s41522-025-00782-y 40715142 PMC12297603

[56] López-Píriz R Sevillano D Fernández Domínguez M Alou L González N Goyos-Ball L. Peri-Implant microbial signature shifts in Titanium, zirconia and ceria-stabilized zirconia reinforced with alumina sites subjected to experimental peri-implantitis: a preclinical study in dogs. Antibiotics. (2024) 13(8):690. 10.3390/antibiotics13080690 39199990 PMC11350813

